# Starvation stress attenuates the miRNA-target interaction in suppressing breast cancer cell proliferation

**DOI:** 10.1186/s12885-020-07118-3

**Published:** 2020-07-06

**Authors:** Jinhui Lü, Chuyi Zhang, Junyi Han, Zhen Xu, Yuan Li, Lixiao Zhen, Qian Zhao, Yuefan Guo, Zhaohui Wang, Evelyne Bischof, Zuoren Yu

**Affiliations:** 1 Research Center for Translational Medicine, Tongji University School of Medicine, 150 Jimo Road, Shanghai, 200120 China; 2 Department of Surgery, Shanghai East Hospital, Tongji University School of Medicine, 150 Jimo Road, Shanghai, 200120 China; 3 Jinzhou Medical University, Liaoning, China; 4 Shanghai University of Medicine and Health Sciences Clinical Medicine Division, Shanghai, China; 5 Division of Internal Medicine, University Hospital of Basel, Petersgraben 4, 4051 Basel l, Switzerland

**Keywords:** Breast cancer, Starvation, miRNA, Target interaction, Proliferation

## Abstract

**Background:**

Emerging evidence has demonstrated the limited access to metabolic substrates as an effective approach to block cancer cell growth. The mechanisms remain unclear. Our previous work has revealed that miR-221/222 plays important role in regulating breast cancer development and progression through interaction with target gene p27.

**Results:**

Herein, we determined the miRNA-mRNA interaction in breast cancer cells under induced stress status of starvation. Starvation stimulation attenuated the miR-221/222-p27 interaction in MDA-MB-231 cells, thereby increased p27 expression and suppressed cell proliferation. Through overexpression or knockdown of miR-221/222, we found that starvation-induced stress attenuated the negative regulation of p27 expression by miR-221/222. Similar patterns for miRNA-target mRNA interaction were observed between miR-17-5p and CyclinD1, and between mR-155 and Socs1. Expression of Ago2, one of the key components of RNA-induced silencing complex (RISC), was decreased under starvation-induced stress status, which took responsibility for the impaired miRNA-target interaction since addition of exogenous Ago2 into MDA-MB-231 cells restored the miR-221/222-p27 interaction in starvation condition.

**Conclusions:**

We demonstrated the attenuated interaction between miR-221/222 and p27 by starvation-induced stress in MDA-MB-231 breast cancer cells. The findings add a new page to the general knowledge of negative regulation of gene expression by miRNAs, also demonstrate a novel mechanism through which limited access to nutrients suppresses cancer cell proliferation. These insights provide a basis for development of novel therapeutic options for breast cancer.

## Background

Breast cancer is still one of the most prevalent neoplasms globally, with a persistently high prevalence despite numerous diagnostic progresses and prevention measures. The mortality is still significant, despite a slight decline in the last two decades. Old age is the most important risk factor for breast cancer, which explains the persistently increasing incidence, especially in developed countries [1–3].

Breast cancer is one of the most heterogenous diseases. Albeit there is a number of established predictive and prognostic factors, most of which are also therapeutic targets (e.g. estrogen, progesterone receptor (ER, PR) or HER-2), the management options are still primarily based on the cancer’s basic clinicopathological features, such as tumor size, lymph node stage, histological grade, type, and lymphovascular invasion [4–7]. Especially, the oldest old cancer patients are being excluded from various therapeutic options due to their frailty and vulnerability towards anticancer-therapy-related side effects [8–10]. Increasingly, discoveries in breast cancer genomics, proteomics and lipidomics are opening our understanding and categorizations [11–13]. However, these analyses are relatively expensive and remain mostly in the theoretical frame. Therefore, there is an urgent need for new indicators and approaches in personalized therapies, offering targeted care with minimal toxicities.

While caloric restrictions in humans showed positive clinical influence on various neurocognitive disorders (NCD) and aging, fasting in cancer patients is controversially debated, especially due to potential increase of tumor growth due to reactive increase of growth factors after fasting cessation, hyperglycemia and hyperinsulinemia, but no valid and reliable trials were conducted so far. Emerging evidence showed starving tumor cells of nutrients are capable to stop cancer cell growing [14]. The constitutive anabolism of cancer cells not only supports proliferation but also addicts tumor cells to a steady influx of exogenous nutrients. Limiting access to metabolic substrates is effective to block cancer growth [15]. Recent reports from animal models illustrated positive relations between starvation and cancer, such as better outcomes in mice after 2–3 fasting days prior to chemotherapy [16–18]. Related hypotheses suggest that fasting renders tumor cells susceptible towards chemotherapy due to a differential stress resistance: since tumor cells are supposedly less adaptable to acute environmental changes, they become hyperactive and hypermitotic under starvation [19]. Such processes lead to chromosomal damage, DNA- and cellular instability, thus a higher sensitivity to chemo- or radio- therapy, despite the fact that paradoxically, these pathophysiologic processes are also causative of tumor growth and progression [20, 21].

Therefore, our study aimed to elaborate on the underlying molecular mechanisms of how fasting links to shrinkage of solid tumors and whether these findings can be translated to humans, using miRNAs (microRNAs) as crucial players in tumorigenesis and progression. miRNAs become severely deregulated during cancerous processes, which impairs their main function on targeted genes by degradation of mRNA or inhibition of mRNA translation. Recently, miRNA-mRNA interactions have been described in the context of signature interactions within well-established cancer pathways, e.g. in breast and pancreatic cancer [22–24]. Nevertheless, some studies revealed that miRNAs have the capability of activating gene expression directly or indirectly in respond to different cell types and cell conditions [24, 25]. However, it is thus far unclear whether miRNA-mRNA interactions depend on the cell status, such as stress induced by external conditions (e.g. starvation). Establishing such correlation would allow for new therapeutic options.

In our previous research, we showed that miR-221/222 is a potential oncogenic gene in breast cancer, and validated target genes p27 downstream of miR-221/222 [26]. The expression levels of miR-221/222 are upregulated in breast cancer, especially in basal-like breast cancer subtype. For example, miR-221/222 showed ~ 20–40 times higher in expression in MDA-MB-231 cell, compared to luminal breast cancer cell types [26]. Targeted knockdown of miR-221 and/or miR-222 suppressed cell proliferation and promoted the cell sensitivity to chemotherapy in human breast cancer [26]. Herein we found that the interaction between miR-221/222 and p27 is dependent on the cell status in human breast cancer cells. Starvation-induced stress showed influence of miRNA-mRNA interaction.

## Methods

### Cell lines and culture

Human breast cancer cell lines MCF-7 and MDA-MB-231 were originally obtained from American Type Culture Collection (ATCC), and maintained in our laboratory. The two cell lines were not included in the list of contaminated cell lines (http://en.wikipedia.org/wiki/List_of_contaminated_cell_lines). Cells are free of mycoplasma contamination according to a recent test in our lab. Cells were regularly cultured in DMEM containing glucose (4.5 g/L), penicillin and streptomycin (100 mg of each/L), supplemented with 10% fetal bovine serum (FBS). Starvation cell culture condition included DMEM containing glucose (1.0 g/L), penicillin and streptomycin (100 mg of each/L) and supplementation with only 0.1% FBS.

### Oligos, plasmid and transfection

Anti-miR-221 (5′ gaaacccagcagacaauguagcu 3′), anti-miR-222 (5′ acccaguagccagauguagcu 3′), anti-miR-NC scramble (5′ guguaacacgucuauacgccca 3′), miR-17-5p (5′ caaagugcuuacagugcagguag 3′), miR-155 (5′ uuaaugcuaaucgugauagggguu 3′) and miR-NC scramble (5′ agucgcauaccucgacaauaau 3′) oligos were designed following LNA Oligo Tools and Design Guidelines of Exiqon (Vedbaek, Denmark), and synthesized per GenScript (Nanjing, China). siRNA to Ago2 and negative control were purchased from Guangzhou RiboBio Co., Ltd. (Guangzhou, China). The HiPerFect transfection reagent from Qiagen (Venlo, The Netherland) was used for RNA oligos cell transfection according to the manufacturer’s instructions. Final RNA oligo concentrations of 50 nM were used for all in vitro assays. miR-221 and miR-222 expression vectors, as previously described in the literature [27], were provided by Moffitt Cancer Center & Research Institute. pcDNA3.1-Ago2 plasmid was presented by Dr. Yandong Li from Tongji University. Lipofectamine 2000 (Invitrogen, USA) was used for plasmid transfections, following the manufacturer’s instructions. Sequences for all primers are available upon request.

### Cell proliferation assays

For the 3-(4,5-dimethylthiazol-2-yl)-2,5 -diphenyltetrazolium (MTT) assay, 4 × 10^3^ cells/well were seeded into 96-well plate in triplicates. After culturing for 24 and 48 h, cells were stained with MTT solution for 3 h under cell-culturing conditions, followed by dissolving with DMSO. The cell growth was determined by measuring OD value at 570 nm wavelength.

### Western blot

Cell lysates (50 μg) were separated by 10% SDS/PAGE. The proteins were transferred to nitrocellulose membrane. After being blocked in 5% milk (w/v) at room temperature for 1 h, the membranes were incubated at 4 °C overnight with primary antibodies (1:1000). Following 1 × PBST washing, the membranes were incubated with secondary antibodies (1:3000) at room temperature for 1 h followed by ECL staining. The following antibodies were applied: anti-p27 (sc-776, Santa Cruz), anti-Cyclin D1 (sc-20,044, Santa Cruz), anti-E2F1 (sc-251, Santa Cruz), anti-Ago2 (04–642, Millipore), anti-Socs1 (ab62584, Abcam) and anti-β-actin (sc-47778, Santa Cruz).

### Luciferase reporter assay

pGL3-p27 3’UTR vector, carrying the whole 3’UTR sequence of human p27 mRNA, was prepared as described in the literature [26]**.** 293 T cells were seeded on 12-well plates at a density of 1 × 10^5^ cells/well in an antibiotic-free medium for 24 h under regular or starvation conditions. 24 h later, 1.0 μg/well of pGL3-p27 3’UTR, 0.2 μg/well of Renilla plasmid and anti-miRNA (50 nM) were co-transfected using HiPerFect transfection reagent from Qiagen (Venlo, The Netherland). For the following 18 h, luciferase activities were measured using Dual-Luciferase Reporter Assay System (Promega) by AutoLumat**.**

### Statistical analysis

The standard two-tailed student’s *t*-test was used for statistical analysis, in which *p* < 0.05 was considered statistically significant. Data are presented as mean ± SEM.

## Results

### Starvation condition suppressed cell proliferation in MDA-MB-231 breast cancer cells

**Fig. 1:**
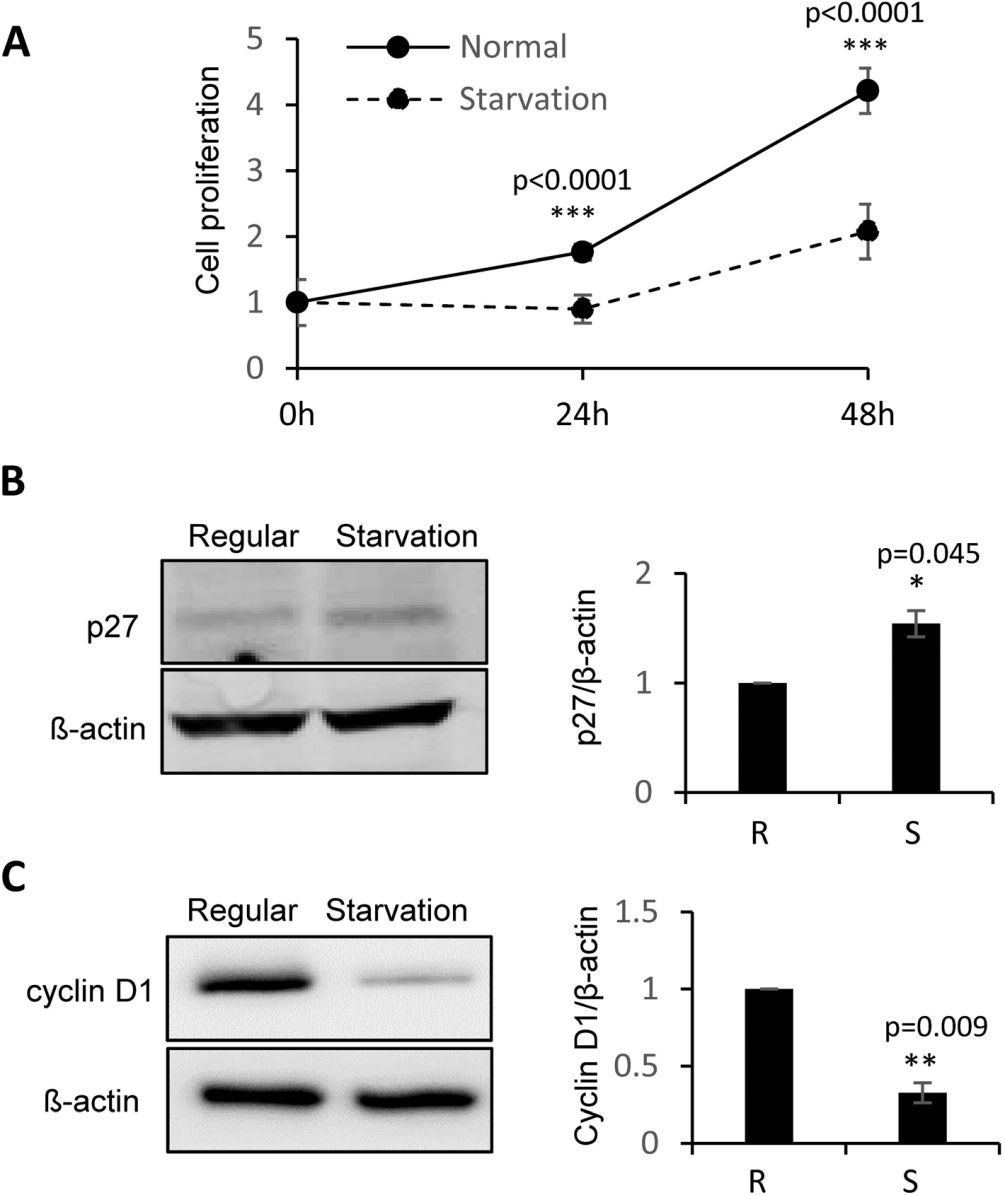
**Starvation stimulation suppressed cell proliferation in human breast cancer. a**: MTT assays showing decreased cell proliferation of MDA-MB-231 cells by starvation stimulation, compared to regular cell culture condition. **b**: Western blot analysis demonstrating the increased p27 expression by starvation stimulation in MDA-MB-231 cells. **c**: Western blot analysis demonstrating the decreased Cyclin D1 expression under starvation in MDA-MB-231 cells. β-actin served as loading control. Original gels for western blots were shown in Supplemental Fig. S6. Data are derived from three independent analyses, and presented as mean ± SEM (*n* = 3). *p < 0.05, **p < 0.01, ***p < 0.0001

In order to determine the influence of the cell proliferation and gene expression by starvation stimulation, MTT assays were conducted in MDA-MB-231 cells under culturing condition of starvation. We observed a significant inhibition of cell proliferation (Fig. 1a). Western blot analysis demonstrated increase of p27 (Fig. 1b) and decrease of Cyclin D1 (Fig. 1c) in expression under starvation condition.

### Starvation-induced stress attenuated the miR-221/222-p27 interaction in MDA-MB-231 breast cancer cells

**Fig. 2:**
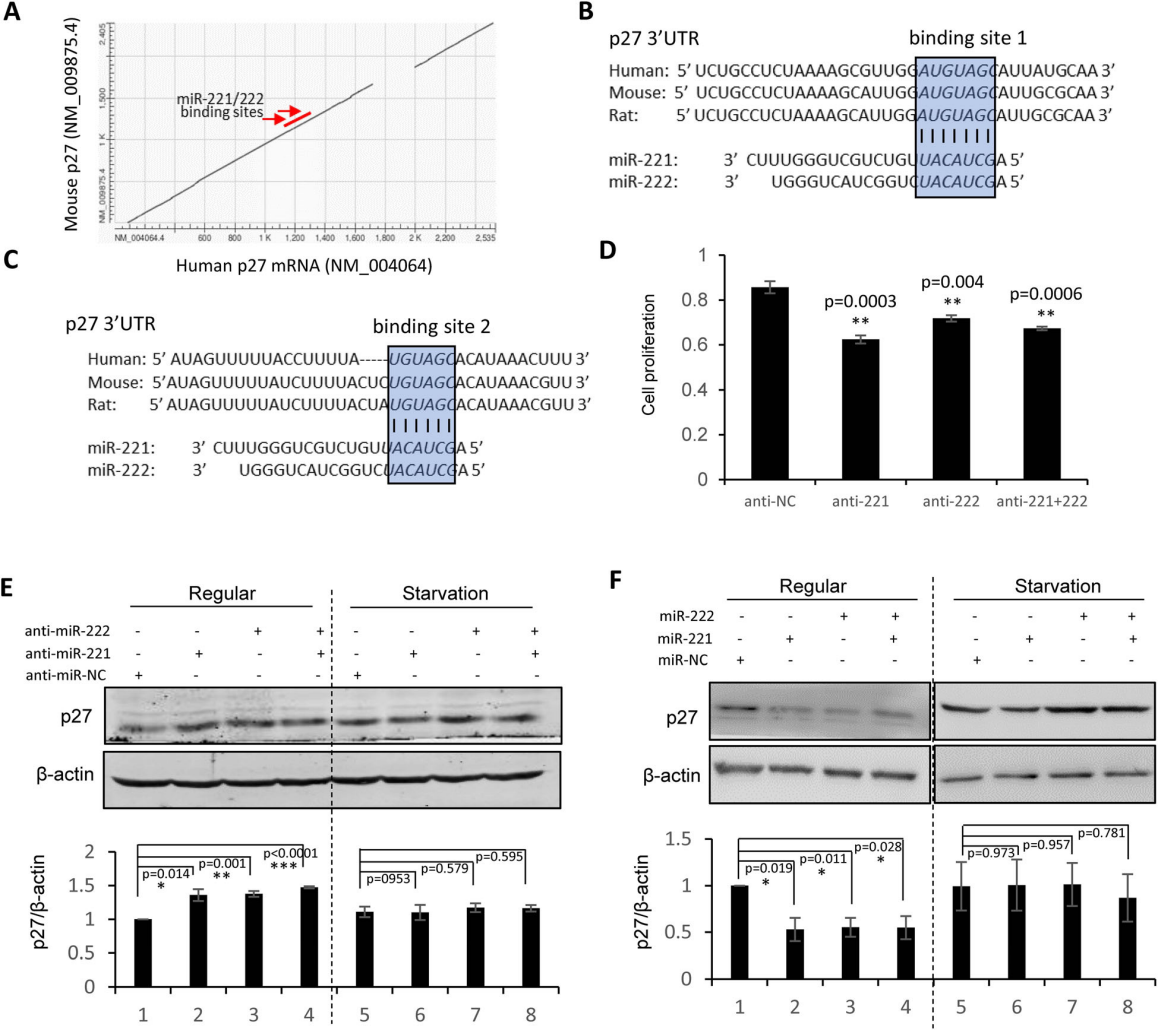
**Starvation stimulation attenuated the miR-221/222-p27 target interaction in regulating cell proliferation in MDA-MB-231. a**: Two binding sites of miR-221/222 were identified in the highly conserved 3’UTR region of p27 mRNAs. **b, c**: Sequence alignment between conserved 3’UTR of p27 mRNA and miR-221/222. The two binding sites were highlighted and boxed. **d**: MTT assays showing decrease of cell proliferation in MDA-MB-231 cells after the knockdown of miR-221 and/or miR-222, compared to negative control (anti-NC). **e**: Western blot analysis showing increased p27 protein level after addition of anti-miR-221 and/or anti-miR-222 into MDA-MB-231 cells under regular cell culture condition, but not under starvation condition. **f**: Western blot showing suppression of p27 at the protein level by overexpression of miR-221 and/or miR-222 in MDA-MB-231 cells under regular cell culture condition, but not under starvation condition. β-actin served as loading control. Original gels for western blots were shown in Supplemental Fig. S6. Data are derived from three independent analyses, and presented as mean ± SEM (*n* = 3). *p < 0.05, **p < 0.01, ***p < 0.0001

miRNA has been well defined to interact with target mRNA through seed sequence complementarity. miR-221/222 has two binding sites within the 3’UTR sequence of target gene p27, which is highly conserved between human and rodents (Fig. 2a-c). Adding miRNA inhibitors to MDA-MB-231 led to a suppression of cell proliferation by both anti-miR-221 and anti-miR-222 (Fig. 2d), which were attenuated by starvation stimulation (Supplemental Fig. S1). miR-221/222 were knocked down or overexpressed in MDA-MB-231 cells, followed by western blot analysis and quantitative real-time PCR to detect the p27 expression at both protein and mRNA levels. As shown in Fig. 2e and Supplemental Fig. S2, p27 expression was significantly upregulated after inhibition of miR-221 and/or miR-222 under regular culture condition, while this upregulation was not seen when the cells were cultured under starvation condition. In consistence, overexpression of miR-221 and/or miR-222 significantly downregulated the expression of p27 under regular condition, but not under starvation condition (Fig. 2f and Supplemental Fig. S3).

**Fig. 3:**
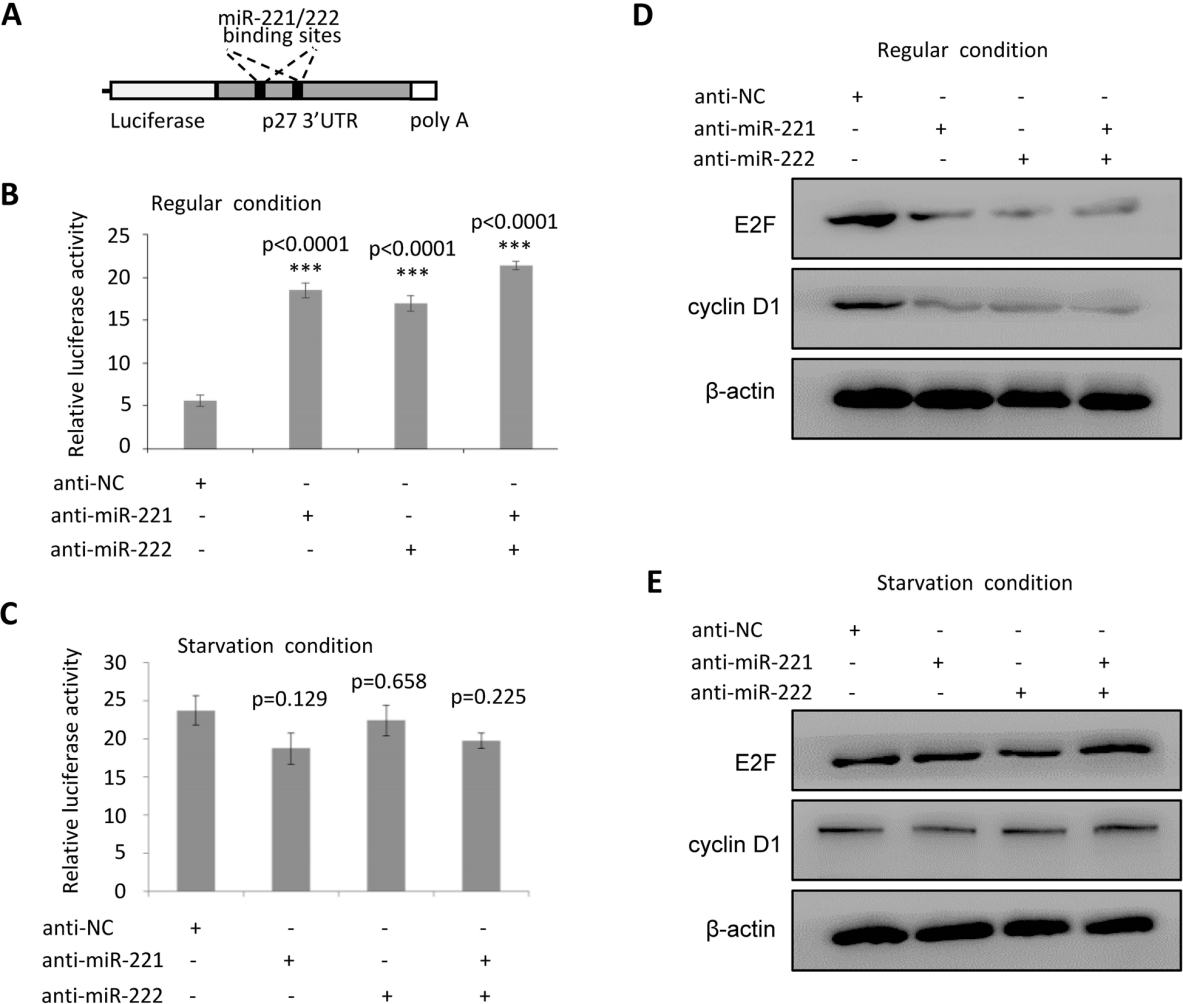
**Luciferase reporter assays demonstrated the attenuated interaction between miR-221/222 and p27 by starvation stimulation. a**: Schematic representation of the luciferase reporter structure carrying human p27 3’UTR. **b**: Luciferase reporter assay demonstrating the direct interaction between p27 3’UTR and miR-221/222 under regular cell culture condition. **c**: Luciferase reporter assay demonstrating the attenuated interaction between p27 3’UTR and miR-221/222 under starvation conditions. **d**: Western blot showing downregulated E2F1 and cyclin D1, two downstream genes of p27, by knockdown of miR-221/222 under normal cell culture condition. **e**: Western blot showing the expression of E2F1 and Cyclin D1 was not affected by miR-221/222 under starvation condition. Original gels for western blots were shown in Supplemental Fig. S6. Data are presented as mean ± SEM (*n* = 4), ***p < 0.0001

In order to further analyze the miRNA-mRNA interaction in cells at starvation-induced stress, a luciferase reporter vector carrying 3’UTR of p27 containing two binding sites to miR-221/222 (Fig. 3a) was transfected into cells. As shown in Fig. 3b, knockdowns of miR-221 and/or miR-222 upregulated the luciferase activity to over 3-fold through interaction with p27 3’UTR under normal cell culture condition. However, the luciferase activity did not show influence by anti-miR221/222 under starvation condition (Fig. 3c), which is consistent with the results in Fig. 2. The expression status of the immediate downstream and associating genes of p27, such as E2F1 and Cyclin D1, was further analyzed under normal and starvation status with or without the presence of miR-221/222. As shown in Fig. 3d, downregulation of E2F1 and Cyclin D1 was associated with upregulation of p27 by knockdown of miR-221/222 under normal cell culture condition. However, this kikd of regulation was not seen under starvation condition (Fig. 3e).

### Other miRNA-target interaction under regular and starvation conditions

**Fig. 4:**
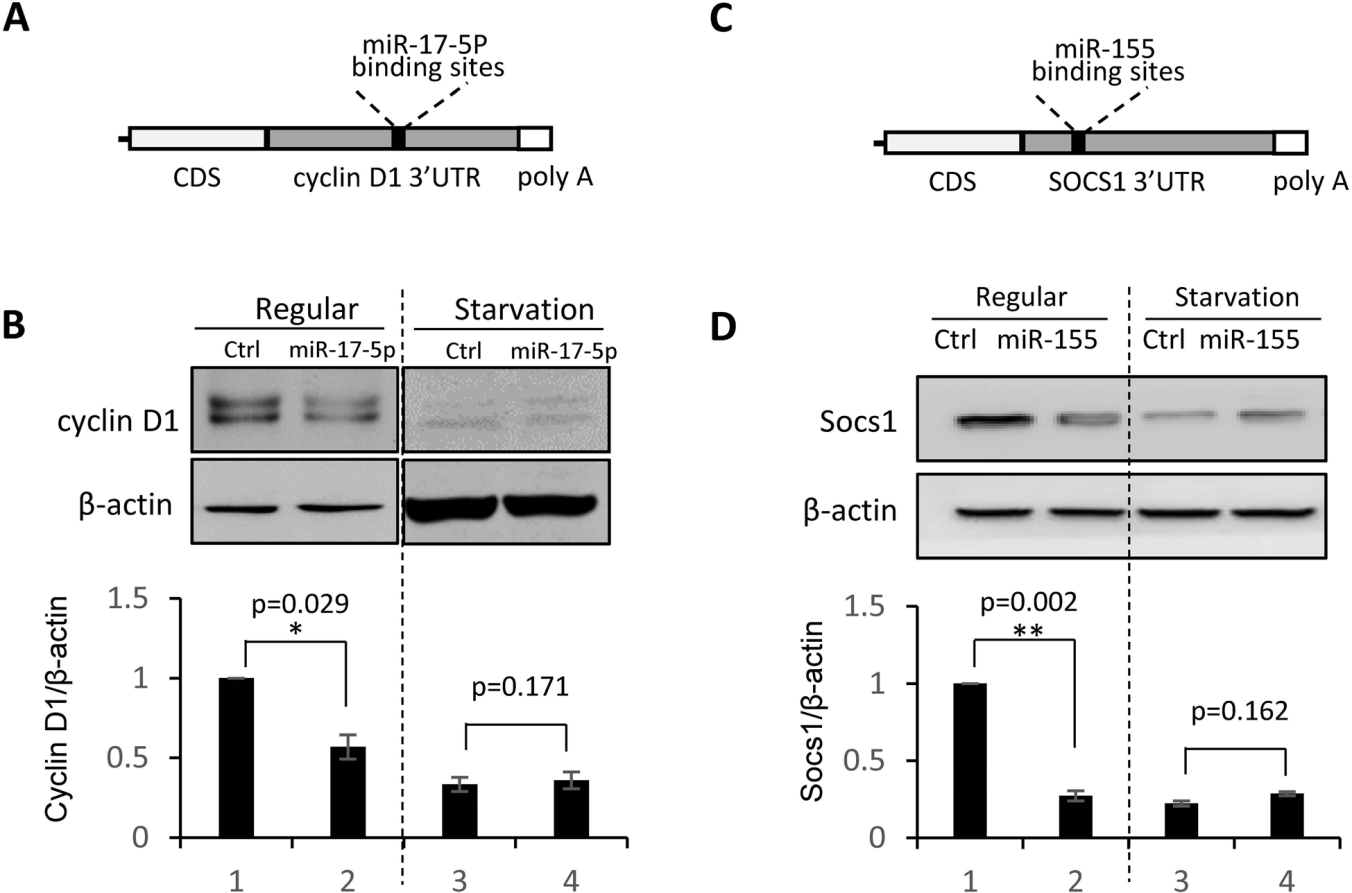
**Other miRNA-target interaction under regular and starvation conditions in breast cancer cells. a**: Sequence binding between 3’UTR of Cyclin D1 mRNA and miR-17-5p. **b**: Western blot analysis showing suppression of the Cyclin D1 expression by miR-17-5p in MCF-7 cells under regular cell culture condition, but not under starvation condition. β-actin served as protein loading control. **c**: Sequence binding between 3’UTR of human Socs1 mRNA and miR-155. **d**: Western blot analysis confirming the miR-155-Socs1 interaction in MDA-MB-231 cells under regular culture condition, but not under starvation condition. Original gels for western blots were shown in Supplemental Fig. S6. Data are derived from three independent analyses, and presented as mean ± SEM (*n* = 3). *p < 0.05, **p < 0.01

In addition to miR-221/222, two more miRNAs with well-defined target gene and regulatory function in human breast cancer were selected to further confirm the influence of the miRNA-mRNA interaction by starvation. As shown in Fig. 4a and b, Cyclin D1 is a demonstrated target gene of miR-17-5p in MCF-7 breast cancer cells [28]. Cyclin D1 expression was significantly lower after transfection with miR-17-5p mimic in MCF-7 cells under normal condition (Fig. 4b). This effect was not observed in cells at starvation-induced stress status (Fig. 4b). Similar analysis was applied to confirmed the suppression of Socs1 by miR-155 in MDA-MB-231 cells under regular culture condition [29], while starvation-induced stress condition completely attenuated this regulation (Fig. 4c-d).

### Decreased Ago2 expression was associated with attenuated miRNA-mRNA interaction under starvation condition

**Fig. 5:**
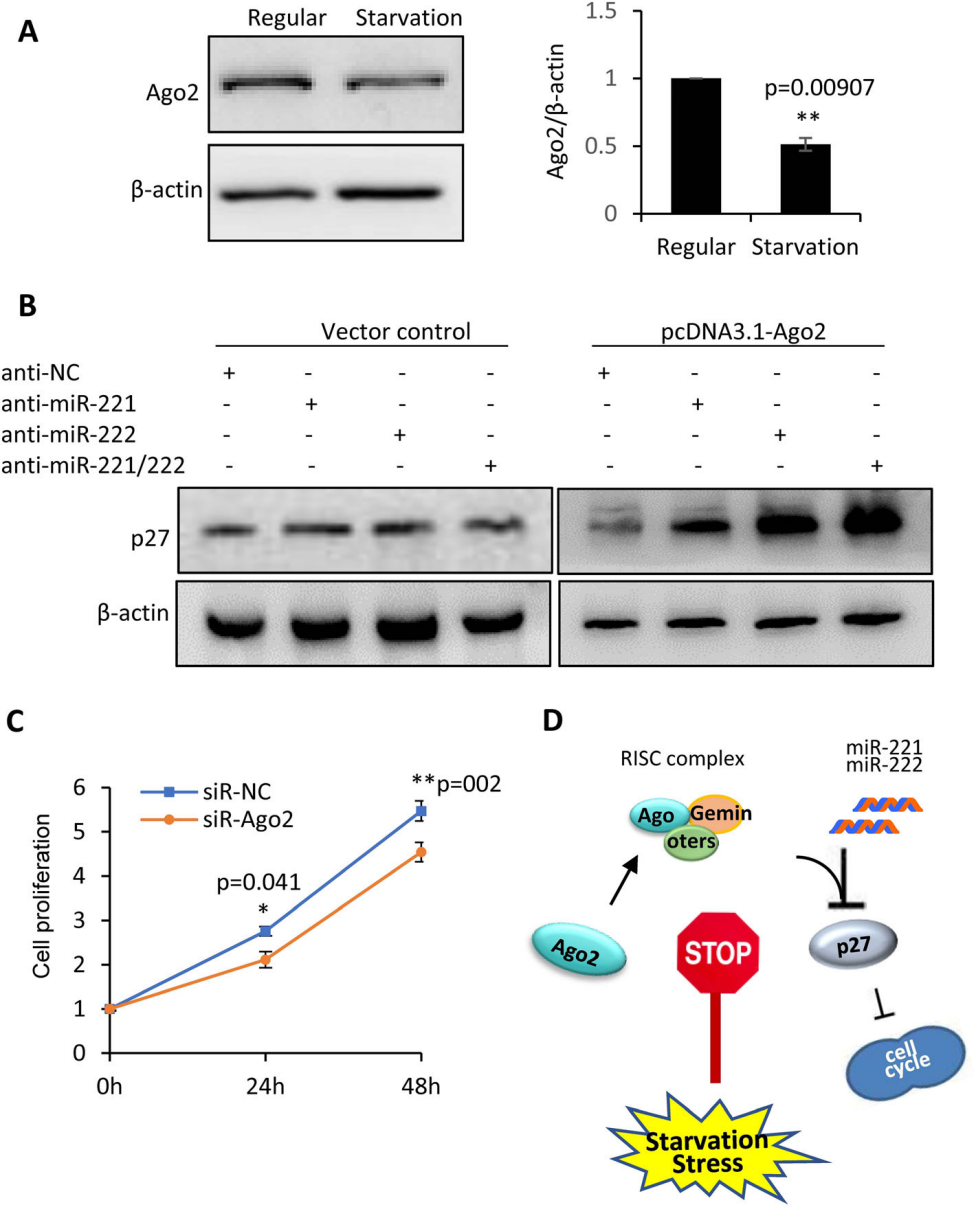
**Suppression of Ago2 expression by starvation stimulation. a**: Western blot analysis demonstrating the inhibition of Ago2 expression by starvation stimulation in MDA-MB-231 cells. β-actin served as loading control. **b**: Western blot analysis demonstrating the attenuated regulation of p27 expression by anti-miR-221 and/or anti-miR-222 under starvation stimulation, which was rescued by addition of Ago2 using pcDNA-3.1-Ago2 plasmid. β-actin served as loading control. Original gels for western blots were shown in Supplemental Fig. S6. **b**: Cell proliferation assay showing decrease of cell proliferation in MDA-MB-231 cells after knockdown of Ago2 by siRNA. **d**: Schematic representation of mechanisms for regulating starvation-induced cell cycle arrest in human breast cancer cells. Downregulation of Ago2 by starvation attenuated the miR221/222-p27 interaction, leading to upregulation of p27 and cell cycle arrest in MDA-MB-231 cells. Data are derived from three independent analyses, and presented as mean ± SEM (*n* = 3). *p < 0.05, **p < 0.01

In order to determine the mechanisms through which miRNA-mRNA interaction is attenuated by starvation stimulation, Ago2 and Dicer1, two key components in the RNA-induced silencing complex (RISC) and regulating the target interaction between miRNAs and mRNAs, as well as Exportin 5 and Drosha, two factors regulating miRNA biogenesis, were detected in MDA-MB-231 cells under regular and starvation conditions. As shown in Supplemental Fig. S4, Ago2 showed downregulation by starvation stimulation, while Dicer1, Drosha and Exportin did not. Western blot analyses were applied to further conform the downregulation of Ago2 by starvation stress (Fig. 5a). The attenuated regulation of p27 expression by anti-miR-221 and/or anti-miR-222 under starvation stimulation was rescued by addition of exogenous Ago2 into MDA-MB-231 cells (Supplemental Fig. S5, Fig. 5b). Targeted knockdown of Ago2 by siRNA treatment in MDA-MB-231 cells led to decreased cell proliferation (Fig. 5c). As such we hypothesized it is the downregulation of Ago2 by starvation that takes responsibility for the attenuated miR221/222-p27 interaction, leading to upregulation of p27 and the cell cycle arrest in MDA-MB-231 cells (Fig. 5d).

## Discussion

In view of the lack of therapeutic target and poor 5-year survival for triple-negative breast cancer, novel forms of therapy are urgently needed. Limited access to nutritious supply is becoming a promising approach for suppressing tumor growth [14, 15]. In addition, miRNA-mRNA-interaction-targeted strategy could enhance the efficacy of chemo- and radio- therapy [30]. While beneficial reactions to fasting have been reported in animals, the underlying pathways have not been fully understood. Herein, we report that starvation suppresses cancer cell proliferation via attenuating miRNA-mRNA target interaction.

In the studied culture of MDA-MB-231 (triple-negative breast cancer cells), the content of miR-221/222 was high [26]. Under normal conditions, it inhibited the expression of p27 and promoted the proliferation and viability of cancer cells. Under starvation, miR-221/222 lost its inhibitory effect on p27 expression, which led to increase of the p27 level and inhibition of the cell cycle. The current study is the first to demonstrate the interruption of miRNA-mRNA interaction by starvation stimulation, which mediates suppression of cell proliferation in breast cancer. Furthermore, Ago2 was decreased under starvation condition, suggesting that RISC structure and function are inhibited in this specific cell status. As such, we propose that the starvation-induced downregulation of Ago2 attenuates the miR-221/222-p27 interaction, leading to a cell cycle arrest in MDA-MB-231 cells. These findings not only demonstrate a novel mechanism for starving cancer cells to inhibit tumor growth, but also add a new page to the general knowledge of the miRNA-mRNA target interaction. It suggests novel therapeutic targets: inhibition of mRNA-miRNA interaction via induced stress, could bring benefits to many breast cancer patients, especially those who lack any therapeutic target or cannot undergo full dose chemo- or radio- therapy.

In addition to miR-221/222-p27 interaction, other miRNA-mRNA interactions, such as miR-155-Socs1 and miR-17-5p-Cyclin D1, were also analyzed, and showed attenuation by starvation stimulation. Socs1, as a member of the suppressor of cytokine signaling family, inhibits Jak/Stat pathway and suppresses cytokine signal transduction. Socs1 is downregulated in breast cancer, functioning as a tumor suppressor [26]. Differing from Socs1, Cyclin D1 is overexpressed in more than 50% of breast cancer, encoding the regulatory subunit of a holoenzyme that phosphorylates the gene retinoblastoma (RB) and promotes G1/S cell cycle progression and oncogenesis [28]. The interrupted miRNA effects on the expression of target genes, such as oncogenes and/or tumor suppressors, should be a mechanism mediating the fasting-induced growth suppression of cancer cells.

In the current study, we demonstrated downregulation of Ago2, a key component of RISC, by starvation stimulation. In view of the similar mechanisms for miRNAs and siRNAs in regulating target gene expression through RISC, siRNA-mediated RNA interference (RNAi) effects on gene expression are very likely influenced in a starvation state. Although further experimental validation is required for the RNAi disruption by starvation stimulation, it remains unclear whether the RNAi strategy can be combined with the fasting strategy for cancer treatment. The implications of disturbed RNAi on the transcriptome in a starvation state have to be taken into account.

## Conclusion

The current study demonstrated the attenuated interaction between miR-221/222 and p27 and decreased Ago2 expression by starvation-induced stress in MDA-MB-231 breast cancer cells. It adds a new page to the general knowledge of negative regulation of gene expression by miRNAs, also demonstrate a novel mechanism through which limited access to nutrients suppresses cancer cell proliferation.

## Supplementary information

**Additional file 1 Figure S1**: MTT assays showing uninfluenced cell proliferation in MDA-MB-231 cells by knockdown of miR-221 and/or miR-222 under starvation condition. **Figure S2**: Quantitative real-time PCR analysis showing increase of p27 expression at mRNA level by anti-miR-221 and/or anti-miR-222 under regular cell culture condition, but not under starvation culture condition. Data are derived from three independent analyses, and presented as mean ± SEM (*n* = 3). **p < 0.01. **Figure S3**: Quantitative real-time PCR analysis showing suppressed expression of p27 at the mRNA levels in MDA-MB-231 cells by miR-221 and/or miR-222 overexpression under regular cell culture condition, but not under starvation condition. Data are derived from three independent analyses, and presented as mean ± SEM (n = 3). **p < 0.01. **Figure S4**: Quantitative real-time PCR analysis of the key factors regulating miRNA biogenesis and function, including Exportin 5, Dicer 1, Ago2 and Drosha in MDA-MB-231 cells under regular and starvation culture conditions. Data are derived from three independent analyses, and presented as mean ± SEM (*n* = 3). **p < 0.01. **Figure S5**: Western blot analysis demonstrating the overexpression of Ago2 in MDA-MB-231 cells transfected with pcDNA 3.1-Ago2 plasmid. Empty vector was used as negative control. β-actin served as loading control. **Figure S6**: Original gels for all western blots in Figures.

## Data Availability

Data sharing is not applicable to this article as no datasets were generated or analyzed during the current study.

## Abbreviations

miRNA: microRNA
RISC: RNA-induced silencing complex
ER: Estrogen receptor
PR: Progesterone receptor
NCD: Neurocognitive disorders
FBS: Fetal bovine serum
RNAi: RNA interference
RB: Retinoblastoma
