# Supplementary material for: Starvation stress attenuates the miRNA-target interaction in suppressing breast cancer cell proliferation
Source: BMC Cancer. 2020 Jul 6;20:627. doi: 10.1186/s12885-020-07118-3 (PMC7339532; doi:10.1186/s12885-020-07118-3)

Supplemental Figure Legends

S1: MTT assays showing uninfluenced cell proliferation in MDA-MB-231 cells by knockdown of miR-221 and/or miR-222 under starvation condition.

S2: Quantitative real-time PCR analysis showing increase of p27 expression at mRNA level by anti-miR-221 and/or anti-miR-222 under regular cell culture condition, but not under starvation culture condition. Data are derived from three independent analyses, and presented as mean ± SEM (n=3). **p<0.01.

S3: Quantitative real-time PCR analysis showing suppressed expression of p27 at the mRNA levels in MDA-MB-231 cells by miR-221 and/or miR-222 overexpression under regular cell culture condition, but not under starvation condition. Data are derived from three independent analyses, and presented as mean ± SEM (n=3). **p<0.01.

S4: Quantitative real-time PCR analysis of the key factors regulating miRNA biogenesis and function, including Exportin 5, Dicer 1, Ago2 and Drosha in MDA-MB-231 cells under regular and starvation culture conditions. Data are derived from three independent analyses, and presented as mean ± SEM (n=3). **p<0.01.

S5: Western blot analysis demonstrating the overexpression of Ago2 in MDA-MB-231 cells transfected with pcDNA 3.1-Ago2 plasmid. Empty vector was used as negative control. β-actin served as loading control.

S6: Original gels for all western blots in Figures.

Supplemental Figures

S1

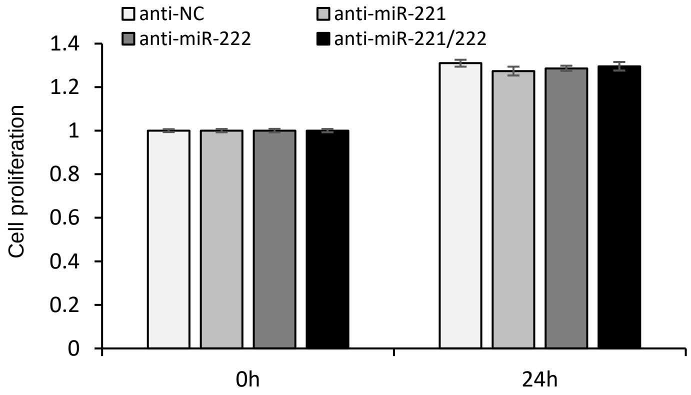

Supplemental Figures

S2

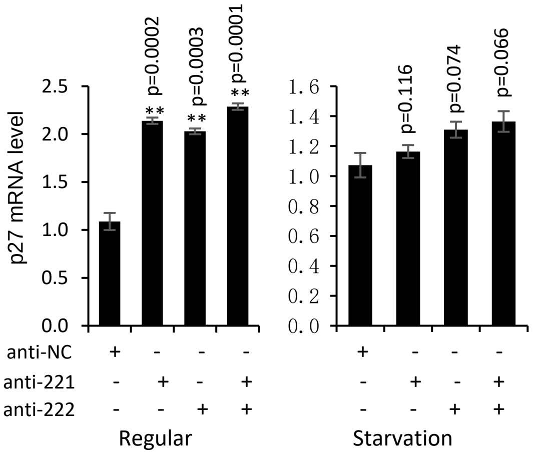

Supplemental Figures

S3

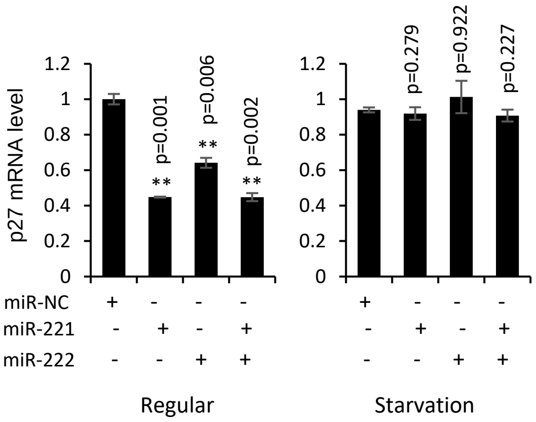

Supplemental Figures

S4

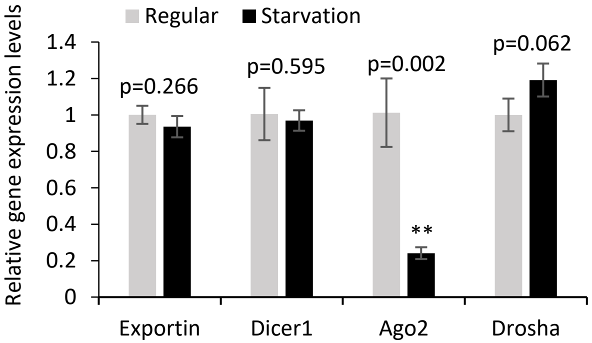

Supplemental Figures

S5

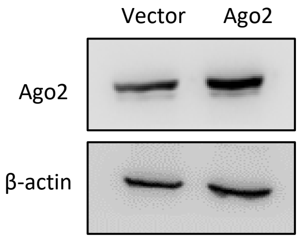

Supplemental Figure S6

Original gel for Figure 1B

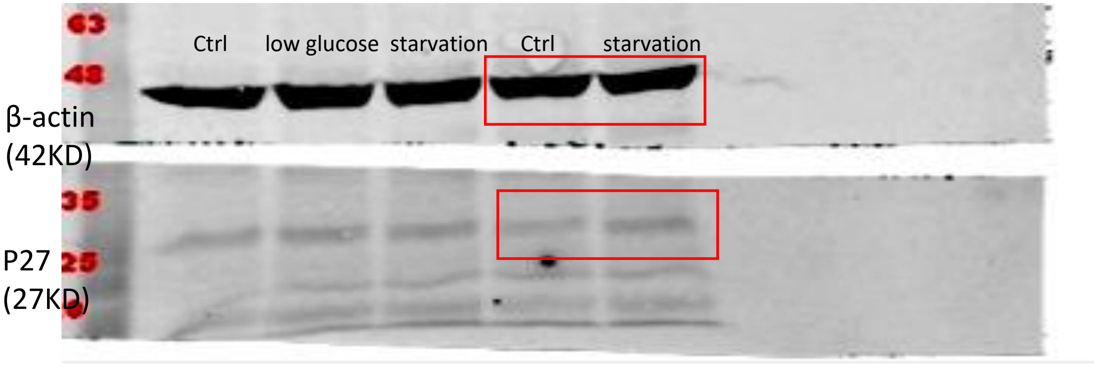

Original gel for Figure 1C

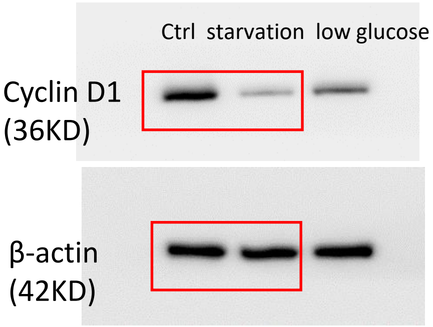

Supplemental Figure S6

Original gel for Figure 2E

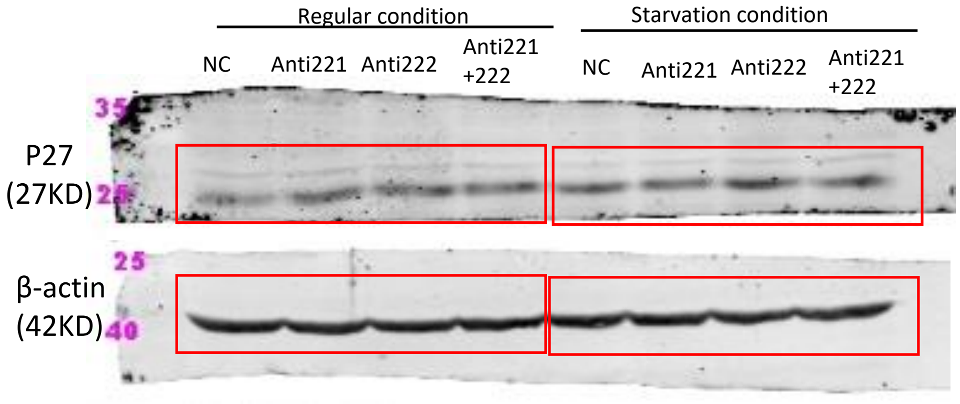

Original gel for Figure 2F

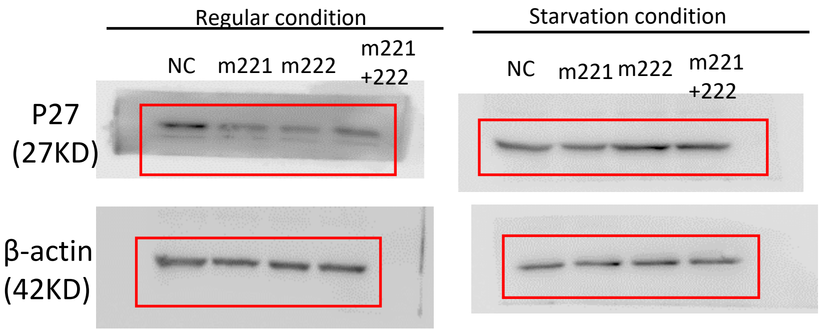

Supplemental Figure S6

Original gel for Figure 4B

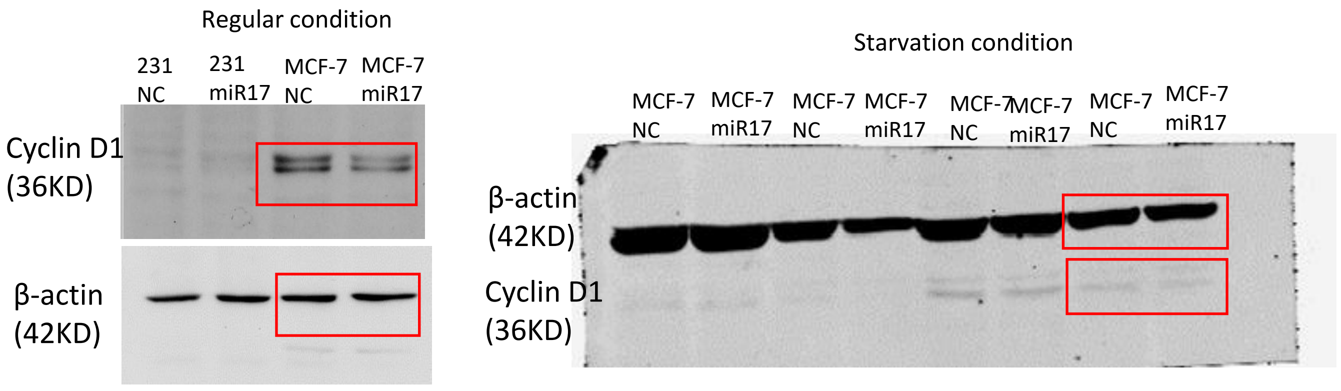

Original gel for Figure 4D

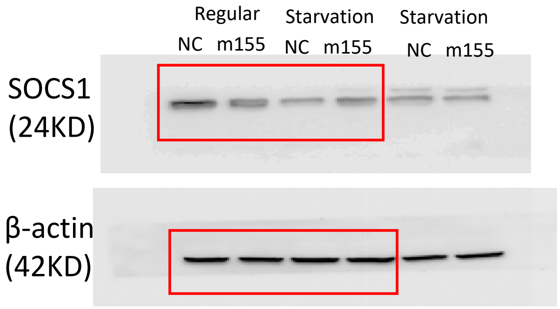

Supplemental Figure S6

Original gel for Figure 3D-E

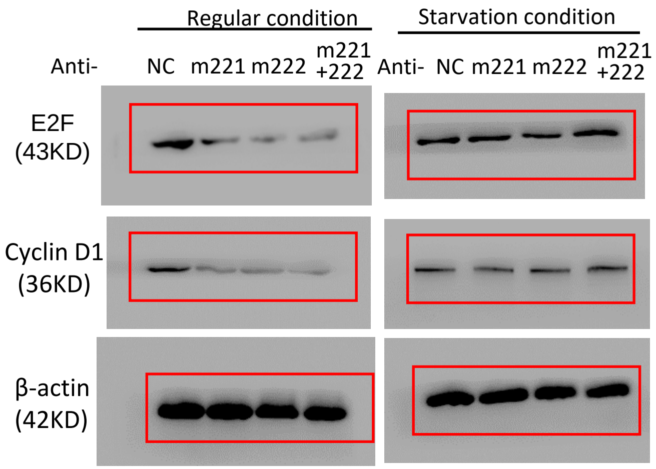

Original gel for Figure 5A

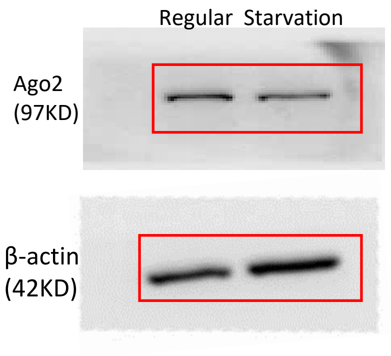

Original gel for Figure S5

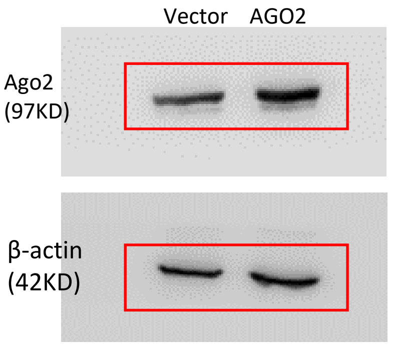

Original gel for Figure 5B

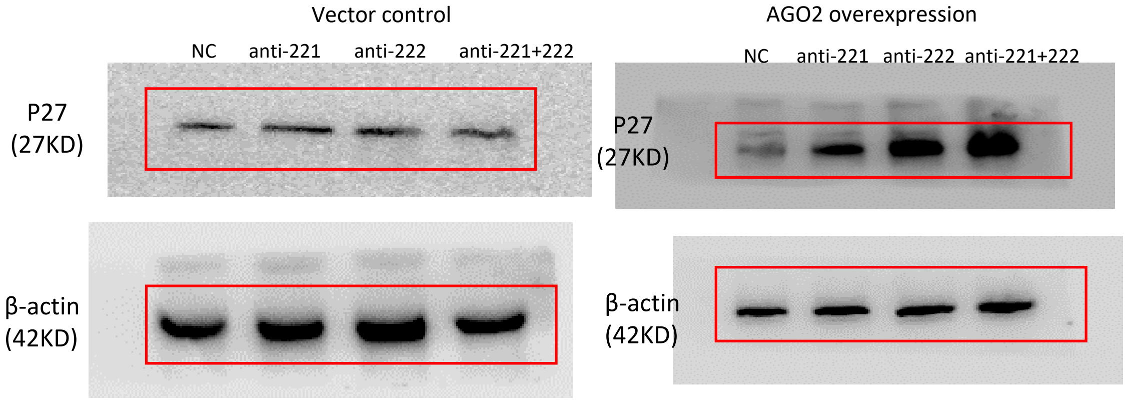

Supplement: Supplementary file 1 — Additional file 1 Figure S1: MTT assays showing uninfluenced cell proliferation in MDA-MB-231 cells by knockdown of miR-221 and/or miR-222 under starvation condition. Figure S2: Quantitative real-time PCR analysis showing increase of p27 expression at mRNA level by anti-miR-221 and/or anti-miR-222 under regular cell culture condition, but not under starvation culture condition. Data are derived from three independent analyses, and presented as mean ± SEM (n = 3). **p < 0.01. Figure S3: Quantitative real-time PCR analysis showing suppressed expression of p27 at the mRNA levels in MDA-MB-231 cells by miR-221 and/or miR-222 overexpression under regular cell culture condition, but not under starvation condition. Data are derived from three independent analyses, and presented as mean ± SEM (n = 3). **p < 0.01. Figure S4: Quantitative real-time PCR analysis of the key factors regulating miRNA biogenesis and function, including Exportin 5, Dicer 1, Ago2 and Drosha in MDA-MB-231 cells under regular and starvation culture conditions. Data are derived from three independent analyses, and presented as mean ± SEM (n = 3). **p < 0.01. Figure S5: Western blot analysis demonstrating the overexpression of Ago2 in MDA-MB-231 cells transfected with pcDNA 3.1-Ago2 plasmid. Empty vector was used as negative control. β-actin served as loading control. Figure S6: Original gels for all western blots in Figures. [file 12885_2020_7118_MOESM1_ESM.pdf]
